# A novel range-verification method using ionoacoustic wave generated from spherical gold markers for particle-beam therapy: a simulation study

**DOI:** 10.1038/s41598-019-38889-w

**Published:** 2019-03-08

**Authors:** Taisuke Takayanagi, Tomoki Uesaka, Masanori Kitaoka, Mehmet Burcin Unlu, Kikuo Umegaki, Hiroki Shirato, Lei Xing, Taeko Matsuura

**Affiliations:** 1 Graduate School of Biomedical Science and Engineering, Hokkaido University, North-13 West-8, Kita-ku, Sapporo, Hokkaido 060-8628 Japan; 2 Hitachi Ltd., 1-1 7-chome, Omika-cho, Hitachi-shi, Ibaraki 319-1292 Japan; 3 Global Station for Quantum Medical Science and Engineering, Global Institution for Collaborative Research and Education (GI-CoRE), Hokkaido University, Sapporo, 060-8648 Japan; 4 Department of Physics, Bogazici University, Bebek, Istanbul 34342 Turkey; 5 Department of Radiation Oncology, Division of Medical Physics, Stanford University School of Medicine, Stanford, CA USA; 6 Faculty of Engineering, Hokkaido University, North-13 West-8, Kita-ku, Sapporo, Hokkaido 060-8628 Japan; 7 Proton Beam Therapy Center, Hokkaido University Hospital, North-15 West-7, Kita-ku, Sapporo, Hokkaido 060-8638 Japan

## Abstract

This study proposes a novel alternative range-verification method for proton beam with acoustic waves generated from spherical metal markers. When proton beam is incident on metal markers, most of the resulting pressure waves are confined in the markers because of the large difference in acoustic impedance between the metal and tissue. However, acoustic waves with frequency equal to marker’s resonant frequency escape this confinement; the marker briefly acts as an acoustic transmitter. Herein, this phenomenon is exploited to measure the range of the proton beam. We test the proposed strategy in 3-D simulations, combining the dose calculations with modelling of acoustic-wave propagation. A spherical gold marker of 2.0 mm diameter was placed in water with a 60 MeV proton beam incident on it. We investigated the dependence of pressure waves on the width of beam pulse and marker position. At short beam pulse, specific high-frequency acoustic waves of 1.62 MHz originating from the marker were observed in wave simulations, whose amplitude correlated with the distance between the marker and Bragg peak. Results indicate that the Bragg peak position can be estimated by measuring the acoustic wave amplitudes from the marker, using a single detector properly designed for the resonance frequency.

## Introduction

Spot-scanning proton therapy (SSPT) is an advanced form of proton therapy, and is being widely employed in newly constructed treatment centers^1^. By superposing many individual spot doses from the Bragg peaks of proton beam, SSPT can confine the radiation dose to the tumour while sparing the surrounding normal tissue^2,3^. However, the precision of the treatment plan is limited by uncertainty in the range of the proton beam and the patient setup. These uncertainties can offset the advantage gained by the spot-scanning technique^4^.

Uncertainty in the range of the proton beam arises from multiple sources, including computed tomography (CT) number to stopping-power conversions and anatomical changes during radiation treatment. These uncertainties have been estimated to about 3.5% of the beam range^5^. Several *in vivo* techniques have been proposed to reduce the uncertainty in the range of proton delivery. Examples include positron-emission tomography (PET)^6^, prompt gamma-ray (PG) detection^7^ and detection of ionoacoustic wave, i.e., acoustic signals originating from the impact of proton beams^8^. PET and PG detection estimate the proton range by measuring the cross sections of nuclear interactions during or immediately following dose delivery.

In contrast, ionoacoustic detection measures the energy transferred by protons at the Bragg peak^8–20^. Like PET and PG detection, acoustic-signal detection is non-invasive. Additionally, acoustic-signal detection does not require bulky equipment for implementation. So far, several simulation studies have focused on waveform analysis in a simple water medium^14,17–19^ and in prostate- and liver-cancer patients^20^. Experimental observations of acoustic emissions have been performed with a linac^9^, a synchrotron^10–13^, a tandem accelerator^14^, a hospital-based isochronous cyclotron^15^ and a synchrocyclotron^16^, with positive results.

Uncertainty in the patient setup can be reduced by exploiting the image guidance techniques. For this purpose, radiographically visible markers placed within or nearby the tumour are commonly employed to monitor the position of the tumour during radiation therapy^21–23^. For tumours that move with respiratory cycles, the markers are monitored in real-time to control beam delivery^24,25^. A wide range of markers with different shapes and materials (gold, steel and others) are used for this purpose, and are selected depending on the tumour site and irradiation strategy^22^.

This article focuses on a spherical-shaped gold fiducial marker and proposes a novel alternative range verification methodology using the acoustic signal emitted from this marker. Gold’s material properties act as a strong pressure source compared to the surrounding biological tissue when irradiated with protons. However, since the large difference in acoustic impedance between gold and water prevents the pressure waves from propagating outward into the tissue, most of these pressure waves are confined within the marker. Only waves with the resonant frequency of the marker gain enough amplitude to propagate outward into the surrounding tissue. We demonstrated this phenomenon in simulations of acoustic-wave transport to explore how this phenomenon can be applied to beam range verification.

## Results

**Figure 1:**
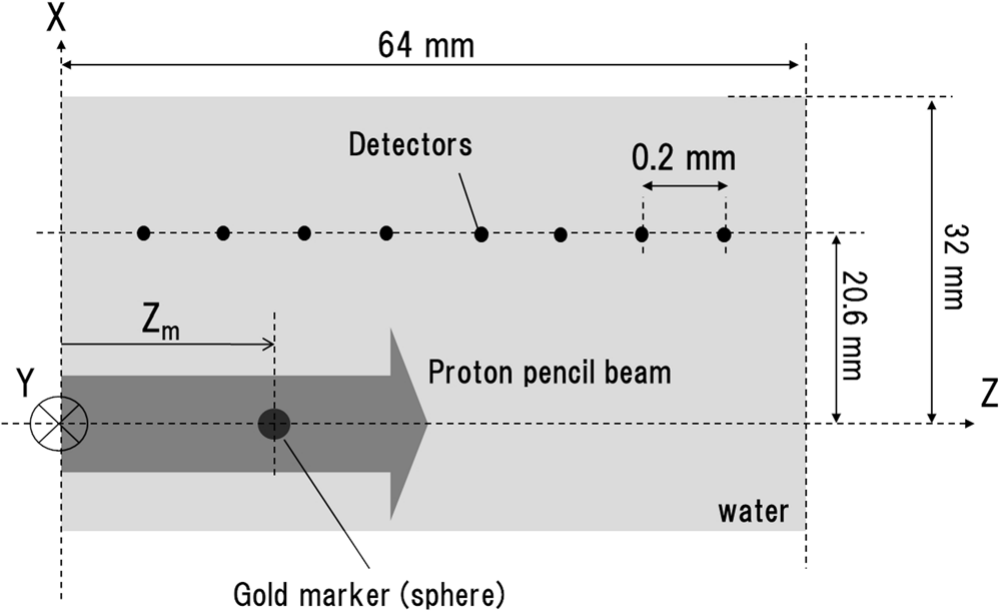
A schematic figure of the simulation environment. The spherical gold marker is placed in a water phantom at depth *Z*_*m*_. Detectors, marked with small black circles, are aligned parallel to the proton beam (grey arrow) at 0.2 mm intervals.

The results discussed below are from three-dimensional simulations that combined dose calculations with a model of acoustic-wave propagation. As shown in Fig. 1, we modelled a spherical gold spherical marker (diameter = 2 mm) placed in water, and a proton pencil beam (incident beam energy = 60 MeV and the spot size = 5 mm) is incident on it along the *z*-axis, which passes through the centre of the marker. The *x* and *y*-axes are taken as the transverse coordinates. To measure the time-resolved pressure waves, point-like detectors are aligned 20.6 mm away from the *z*-axis. The details of the simulation environment are described below in section 4. The pressure waves were measured in both temporal and frequency domains with variations of the following parameters: the beam pulse width *σ*_*p*_ and the marker position *Z*_*m*_.

### General characteristics of transferred energy and waveform

**Figure 2:**
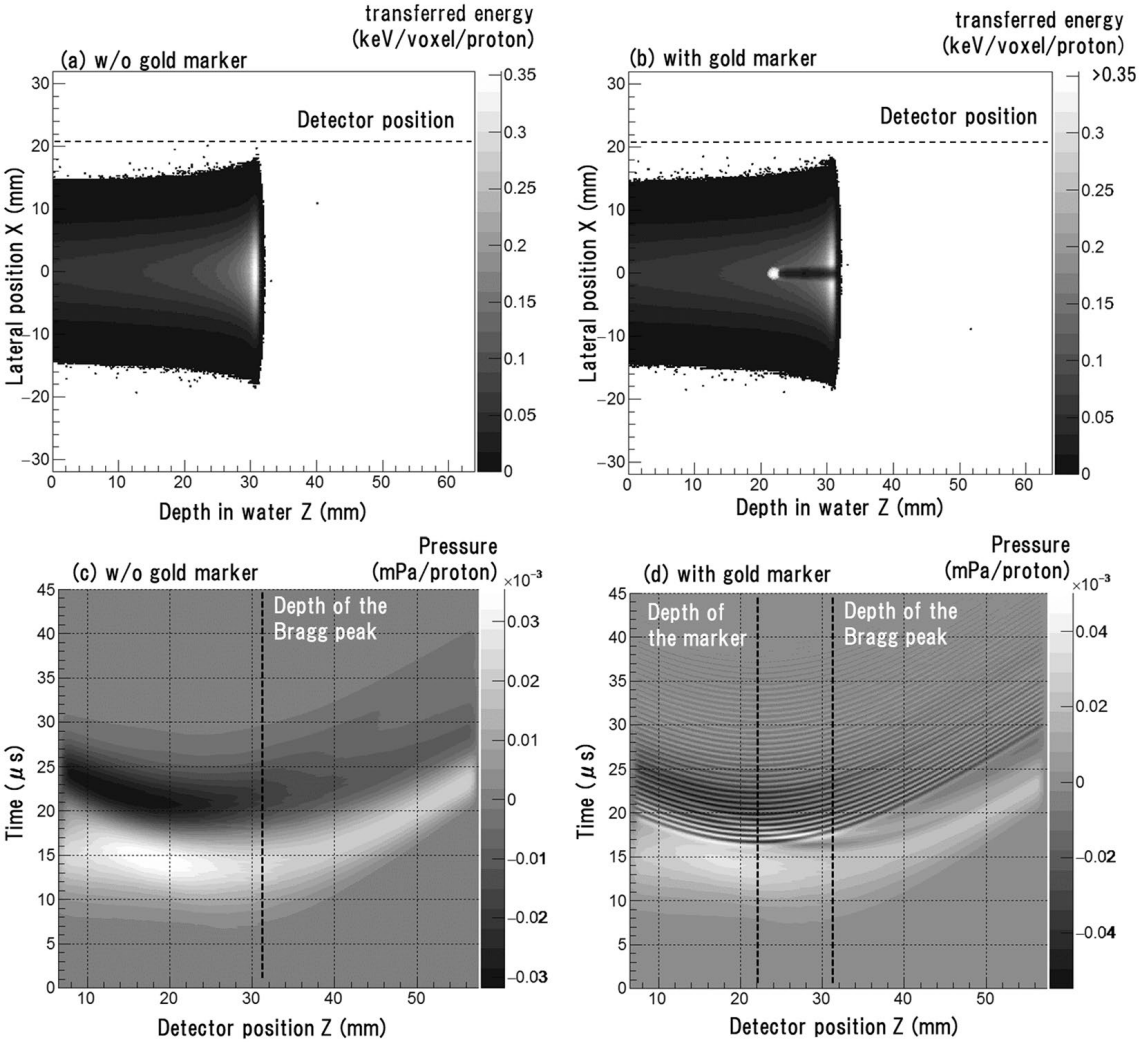
Distribution of transferred energy from proton beam on the *z-x* plane (**a**) without a gold marker (**b**) with a 2.0-mm-diameter gold marker placed at *Z*_*m*_ = 22 mm. The horizontal axis is depth in water and the vertical axis is position along the *x*-axis marked in Fig. 1. Voxel volume = 0.2 × 0.2 × 0.2 mm^3^. (**c**,**d**) are the acoustic wave propagation from the transferred energy distributions displayed in (**a**,**b**), respectively. An incident pulse width is 100 ns. The horizontal axis indicates the detector position, the vertical axis indicates the time from the incident pulse peak, and the greyscale colour indicates the sound pressure.

In this study, we assume that the proton beam is injected in a short period of time (*σ*_*p*_ = 30–500 ns), which is within the output range of fast-extraction synchrotron, fixed-field alternating gradient, dielectric wall and laser-driven accelerators^19^. Figure 2(a,b) show the transferred energy distributions on the *z-x* plane without and with the gold marker, respectively. Protons transfer 9.5 times more energy in gold than they do in water due to gold’s relatively high electron density. Accordingly, a hot spot arises in Fig. 2(b) and a cold region develops downstream of the gold marker.

Figure 2(c,d) show the time-resolved pressure waves as measured along the line of detectors in the absence and presence of the gold marker, respectively (*σ*_*p*_ = 100 ns). As shown in Fig. 2(c), with no gold marker, a typical bipolar waveform appears; the *z*-dependent arrival time at the post-Bragg peak region (*Z* ≳ 31 mm) is a feature of γ waves, while the weaker *z*-dependency of arrival time is a feature of α waves at pre-Bragg peak positions (*Z* ≲ 31 mm)^8^.

As shown in Fig. 2(d), the presence of a gold marker introduces a distinctive high-frequency component to the original waveform. It is symmetric around the depth of the marker (*Z*_*m*_ = 22 mm), and its frequency does not depend on the detector position. The maximum pressure of −5.5 × 10^−5^ mPa/proton is achieved at *Z* = 16.2 mm at *t* = 21.1 μs, and the absolute value of the pressure is about 1.5 times higher than it is with no gold marker present (3.6 × 10^−5^ mPa/proton at *Z* = 17.0 mm and *t* = 14.8 μs).

### Effects of the beam pulse width *σ*_*p*_

In this section, we discuss how the frequency and amplitude of the acoustic wave were affected by changing the pulse width. These simulations were run with the detector placed at the same depth as the marker, *Z* = 22 mm.

**Figure 3:**
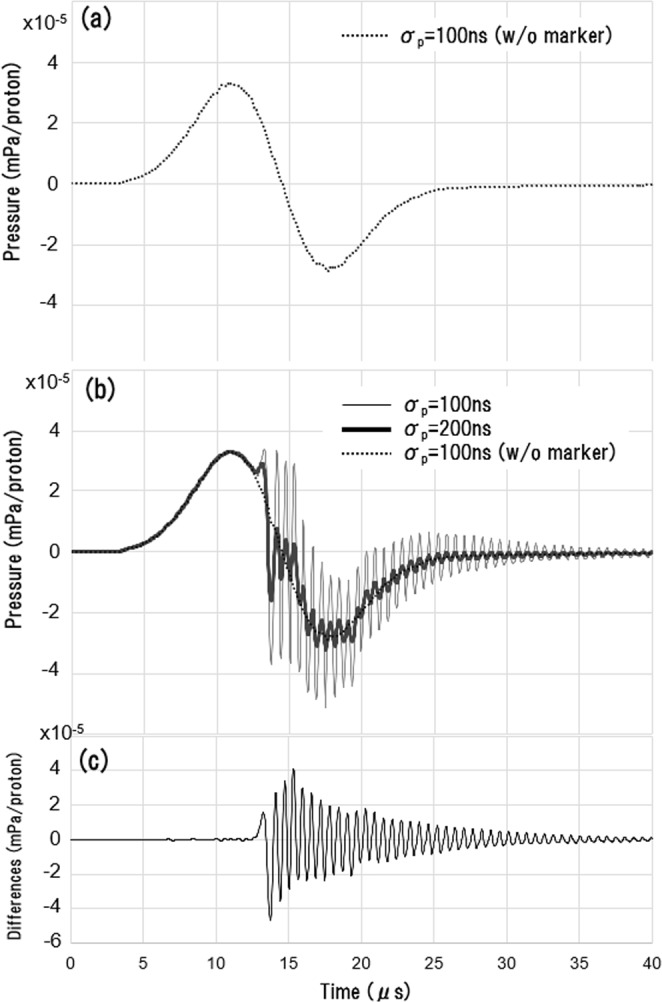
(**a**) Acoustic waveform in the absence of a marker with σ_*p*_ = 100 ns. (**b**) Acoustic wave in the presence of a marker with σ_*p*_ = 100 and 200 ns. The marker is placed at *Z*_*m*_ = 22 mm (same depth as the detector (dotted line in Fig. 2 (d))). (**c**) Difference between the pressures with and without the gold marker. The horizontal axis gives the time (μs) from the incident pulse peak and the vertical axis indicates the pressure (mPa/proton).

Figure 3(a) shows the pressure wave simulated with no marker present, and with an incident pulse width of 100 ns. The waveform has a bipolar shape originating from the dose distribution of the pencil beam. The peak-to-peak time of the bipolar waveform (~6.5 μs) can be deduced from the lateral size of the pencil beam (FWHM ~12 mm), which corresponds to a pressure-wave propagation time of ~8 μs. Since this value was two or three orders of magnitude greater than the range of the tested pulse width (*σ*_*p*_ = 30–500 ns), *σ*_*p*_ has a negligible effect on the waveform.

Figure 3(b) shows the waveform simulated with a gold marker present and with *σ*_*p*_ = 100 ns (thin solid curve) or 200 ns (thick solid curve). The high-frequency wave appears immediately after the first positive peak emitted from the pencil beam. The amplitude of the wave decreases as *σ*_*p*_ increases. Figure 3(c) plots the difference between the waveforms measured with and without the gold marker present. The maximum pressure measured with the gold marker present was −4.7 × 10^−5^ mPa/proton and occurred at *t* = 13.7 μs. This maximum pressure was generated at the gold marker, as its timing is close to the distance between the marker and the detector divided by the speed of sound in water, i.e., 13.1 μs.

**Figure 4:**
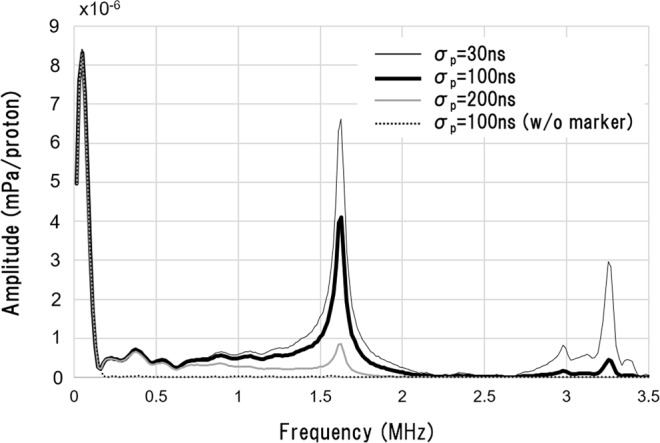
Frequency spectrums of the acoustic waves generated from a proton beam pulse with *σ*_*p*_ = 30, 100 and 200 ns. The horizontal axis is frequency, and the vertical axis is amplitude. Results are shown for a 2-mm-diameter marker at *Z*_*m*_ = 22 mm (same depth as the detector).

Next, a Fourier transform was applied to the pressure waves in Fig. 3(a,b) to examine their frequency distributions, as shown in Fig. 4. With no marker present, the signal energy arising from the pencil beam is concentrated in the low-frequency regime (*f* < 200 kHz). In this regime, the amplitude is independent of *σ*_*p*_, as expected from the discussion above. With a gold marker present, a specific resonance peak appears at 1.62 MHz. The resonant frequency does not depend on *σ*_*p*_, and its magnitude is negatively correlated with *σ*_*p*_. In the cases of *σ*_*p*_ = 30, 100 ns, a second peak appears at 3.24 MHz, which is twice the frequency of the first peak. These values agree with the eigenvalues of the wave equations with zero boundary conditions on the sphere surface, as discussed in section 3 below (*n* = 1 and 2 in Eq. (6), respectively).

### Effects of marker position *Z*_*m*_

The resonance-peak frequency and amplitude were next explored by varying the marker positions from *Z*_*m*_ = 16 to 38 mm. Like above, these simulations were run with the detector placed at the same depth as the marker (*Z* = *Z*_*m*_). As with Fig. 4, two peaks were observed at 1.62 and 3.24 MHz and both were independent of *Z*_*m*_. For the sake of concision, we concentrate on the first resonance peak at 1.62 MHz below.

**Figure 5:**
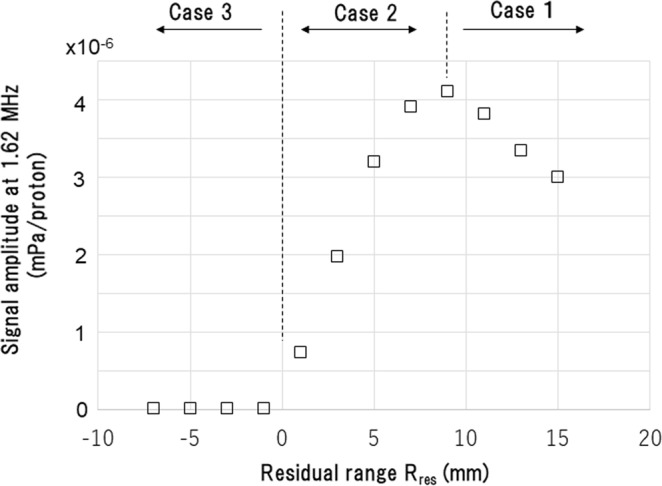
The peak amplitude at the resonant wave at 1.62 MHz in terms of the residual range *R*_*res*_ at marker positions defined by Eq. (1). The vertical axis represents the signal amplitude at 1.62 MHz. Results are shown for *σ*_*p*_ = 100 ns.

In Fig. 5, the amplitude of the resonance peak is plotted in terms of the residual range *R*_*res*_ at marker position, which is defined as follows

1 $${R}_{res}={R}_{0}-{Z}_{m}.$$

Here, *R*_0_ is the proton range, which is 31 mm in this case (incident beam energy = 60 MeV).

Based on the characteristics of the plot in Fig. 5, we categorise the marker positions into three cases: case 1: *R*_*res*_ > 9 mm (marker is placed more than 9 mm upstream of the Bragg peak), case 2: 0 < *R*_*re*s_ ≤ 9 mm (marker is placed less than 9 mm upstream of the Bragg peak), and case 3: *R*_*res*_ ≤ 0 (marker is placed downstream of the Bragg peak).

In case 2, the amplitude of the acoustic wave increases almost linearly with *R*_*res*_ at a rate of approximately 15% per mm_._ In this case, any protons entering the marker transfer all their residual kinetic energy to the marker and are stopped inside the marker. Therefore, the acoustic wave amplitude will be larger if the proton energy is greater, which is the case as the residual range increases. A range verification method that exploits this phenomenon will be discussed in the following section. In case 1, on the other hand, the proton beam passes the marker and the energy transferred to the marker is roughly proportional to the stopping power at *R*_*res*_. As such, the amplitude of the acoustic wave emanating from the gold marker increases as *R*_*res*_ decreases. The wave amplitude is affected less by *R*_*res*_ as *R*_*res*_ increase, reflecting how the proton energy loss decreases slowly at large *R*_*res*_. Finally, no signal appears at all in case 3, since the beam does not reach the gold marker.

## Discussion

Our wave-propagation simulations showed that resonant waves are emitted from the gold marker after the injection of the pulsed proton pencil beam. Here, we analytically derive the properties of this resonant wave that appeared in simulations.

The observed waveform can be decomposed into the waves originating within and outside of the marker. Waves originating outside the marker can be approximated as waves originating from the pencil beam dose distribution with no marker present; although the gold marker develops the cold regions downstream as shown in Fig. 2(b), the area of dose distortion is small enough that it negligibly impacts the acoustic waveform. The rest of the waveform originates within the marker, which acts as a spherically-symmetric heat source. Immediately after proton injection, thermalisation occurs due to the high heat conductivity of gold and the marker reaches a uniform temperature very quickly. During proton injection, a large amount of energy is transferred to the gold (9.5 times the amount energy transferred to water). This energy is rapidly converted to a large pressure wave since the Gruneisen coefficient of gold, i.e., the conversion factor from thermal energy to pressure, is 32 times greater than that of water. However, a large part of the acoustic energy does not reach the surrounding water and is reflected back into the gold as there is a large difference in acoustic impedance at the interface between the gold marker and water. As a result, wave pressure in water reduces to 5% of that in the marker.

Since the acoustic reflectance is nearly 1 at the boundary, let us first assume perfect reflection and set the pressure field $p(\overrightarrow{r},\,t)$ to be zero at the boundary. Considering the spherical symmetry of the system, the pressure field with the frequency *f* can be denote as $p(\overrightarrow{r},\,t)=p(r){e}^{i2\pi ft}$, where *r* is a radial coordinate with its origin located at the centre of the marker. Then, the acoustic wave equation^8,14,26,27^

2 $${\nabla }^{2}p(\overrightarrow{r},\,t)-\frac{1}{{v}^{2}}\frac{{\partial }^{2}p(\overrightarrow{r},\,t)}{\partial {t}^{2}}=0$$

becomes

3 $$\frac{{\partial }^{2}p(r^{\prime} )}{\partial {r^{\prime} }^{2}}+\frac{2}{r^{\prime} }\frac{\partial p(r^{\prime} )}{\partial r^{\prime} }+p(r^{\prime} )=0,$$

where

4 $$r^{\prime} =\frac{2\pi f}{v}r.$$

and *v* is the speed of sound in gold (see Table 1).

**Table 1:** Material properties of water and gold.

	*ρ*(g/cm^3^)	*β*(K^−1^)	*v*(m/s)	*C*_*P*_(J/K/kg)	*Γ*	*Z*(Ns/m^3^)	*α*_*th*_ (m^2^/s)
Water (27 °C)	1	210 × 10^−6^	1500	4180	0.11	1.5 × 10^6^	0.15 × 10^−6^
Gold	19.3	42.6 × 10^−6^	3240	128.8	3.47	6.3 × 10^7^	128 × 10^−6^

*ρ*: mass density, *β*: coefficient of volumetric thermal expansion, *v*: speed of sound, *C*_*P*_: heat capacity at constant pressure, *Γ*: Gruneisen coefficient, *Z*: acoustic impedance, and *α*_*th*_: thermal diffusivity.

Spherical Bessel functions are the solutions of Eq. (3), with the zero-th order expressed as

5 $$p(r^{\prime} )\propto \frac{\sin (r^{\prime} )}{r^{\prime} }.$$

If the diameter of the gold marker is *φ*_*m*_, the boundary condition of complete reflection can be written as $p(\pi f{\phi }_{m}/v)=0$. The resonance frequency *f*_*res*_ is thereby:

6 $${f}_{res}=\frac{v}{{\phi }_{m}}n\,(n:\,{\rm{integer}}).$$

This resonant wave would mostly survive inside of the marker. However, as the reflectance is slightly less than 1 in practice, the resonant wave gradually releases energy to the water, generating the acoustic signal that was observed in the simulations. Pressure waves with frequencies other than *f*_*res*_ will cancel each other out when reflecting within the marker.

**Figure 6:**
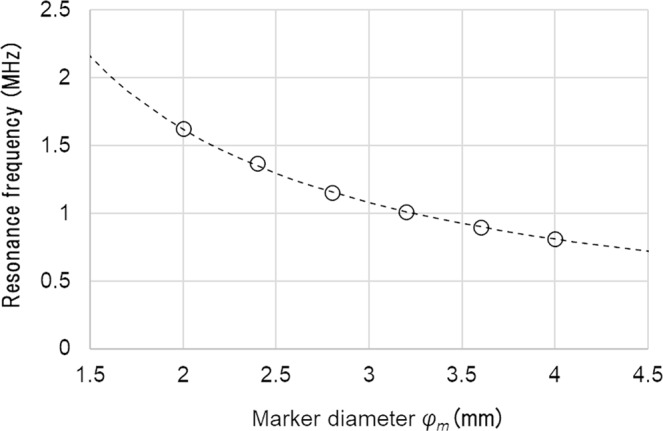
Resonance frequencies of the acoustic waves generated from the gold marker. The horizontal axis is the marker diameter *φ*_*m*_. The simulation results (marked with ○) are plotted along with the theoretical curve calculated by Eq. (6) for n = 1 (dotted curve). The case of σ_p_ = 100 ns and *Z*_*m*_ = 22 mm is shown here. These results were simulated with a detector placed at *Z* = 22 mm, which is at the same depth as the gold marker.

To check the validity of the mechanism described above, the peak frequencies obtained by the simulation for different marker sizes are plotted in Fig. 6. The theoretical prediction (Eq. (6)), plotted in a dotted line, perfectly matches the simulation results.

**Figure 7:**
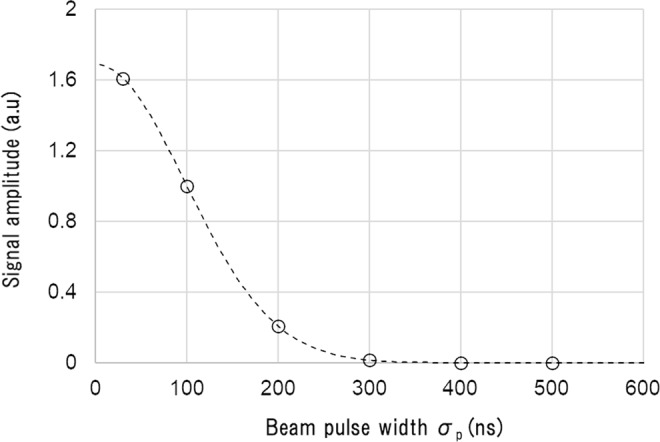
Signal amplitude at the resonance frequency of 1.62 MHz. The horizontal axis is the pulse width σ_p_. The vertical axis is the normalised signal amplitude at σ_p_ = 100 ns. Circles show results obtained from the simulation, and the dotted curve was calculated from Eq. (7). The simulation with φ_m_ = 2.0 mm and *Z*_*m*_ = 22 mm is shown here and the detector is placed at the same depth as the gold marker.

In addition, the above treatment predicts that the resonance-peak amplitude should behave as

7 $$A({\sigma }_{p})\propto \exp \,[\,-\,\frac{{(2\pi {f}_{res}{\sigma }_{p})}^{2}}{2}]$$

with respect to the Gaussian pulse width *σ*_*p*_. As is shown in the Fig. 7, the simulation results for different pulse widths match perfectly with Eq. (7), which again supports the validity of the above analysis.

Range verification is an important potential application of the acoustic wave signal from a gold marker. As Fig. 5 shows, the amplitude of the acoustic wave emanating from the gold marker is correlated with *R*_*res*_, i.e., the distance between the marker and the Bragg peak. Especially, when *R*_*re*s_ is in the range of 0 < *R*_*re*s  _< 9 mm (case 2), the amplitude of the acoustic wave (*A*) is almost linearly related to *R*_*res*_, namely:

8 $${R}_{res}={C}_{1}A+{C}_{0}$$

where *C*_1_ and *C*_0_ are constants that can be derived for each spot by using wave simulations on patient CT image. If these constants are calculated before treatment, *R*_*res*_ can be estimated in real time from measurements of *A* recorded by acoustic transducers. The marker position is measured by X-ray radiography or fluoroscopy^23,25^ and the beam axis is measured by profile monitors installed in the treatment nozzle. Coupled with these data, the acoustic measurements allow the calculation of the absolute position of the Bragg peak within a patient.

Although the range verification method outlined above is applicable only if the marker is placed less than 9 mm upstream of the Bragg peak, a simpler method may be useful if the proton beam stops just before the marker position (|*R*_*res*_| ~ 0; case 3 in Fig. 5). In that case, the appearance of a resonant wave would indicate that the proton range is longer than anticipated. This method will be especially useful if the patient’s situation allows the marker to be inserted at the boundary between the tumour and critical organs, since it could be used as a binary warning to prevent damage to critical organs. To accurately monitor a respiratory-moving tumour, several markers are generally placed around the tumour. If these markers have different diameters, the warned spot could be identified based on the various resonance frequency of the markers.

The range-verification method proposed here is not limited to proton beams alone, but is rather also applicable to other beam-scanning treatments with heavy ions, such as a carbon-ion beams. Once a spot is selected that is suitable for range verification, i.e. 0 < *R*_*re*s < _9 mm (case 2), a high dose should be delivered to those spots to generate a large-amplitude pressure wave, while suppressing the dose to surrounding spots in order to avoid inhomogeneity in the dose. Intensity-modulated proton therapy (IMPT) is therefore the ideal application for this range-verification method.

In contrast to the measurement of the acoustic wave from the Bragg peak, the frequency of the resonant wave from the gold marker depends solely on the marker size; it does not depend on the detector positioning or the properties of the incident beam (i.e., the lateral beam size, the width of the Bragg peak, the incident pulse width etc.). Therefore, the position of the Bragg peak can be estimated with only a single acoustic transducer, if it is properly designed for the resonant frequency of the gold marker.

To experimentally validate the clinical application of the proposed method, we discuss the magnitude of the possible range detection errors due to transducer noise and uncertainties in transducer positioning and beam delivery. For simplicity, we perform estimations with the same conditions as those used to derive the plot shown in Fig. 5 (i.e., a beam energy of 60 MeV, a spot size of 5 mm (1σ), a pulse width of 100 ns, and a marker to detector distance of 20.6 mm), where the linear coefficient *C*_1_ in Eq. (8) is 2.3 × 10^6^ mm · proton/mPa.

Since we obtain the range from the measured acoustic pressure, range precision is limited by thermal noise in the transducer. According to a recent study^18^, the noise level of PZT transducers is typically about 0.03 mPa/$\sqrt{{\rm{Hz}}}$, which indicates that the noise of 35 mPa will be added to the resonant wave of 1.62 MHz. As a result, to detect the range within 1 mm of accuracy, each pulse requires more than 7.8 × 10^7^ particles. Since a range of 3 × 10^8^ to 8 × 10^8^ protons per pulse is required to deliver a 2 Gy dose at the Bragg peak, clinical proton-beam therapy applications should generate sufficiently strong acoustic waves to verify the range. The range detection error increases for higher energy beams with an identical number of protons because higher energy beams have a wider Bragg peak due to energy straggling that causes an increase in the linear coefficient *C*_1_. Discussed later, the SNR should increase in such cases.

The high frequency of the resonant wave, on the order of MHz, will lead to strong attenuation in the tissue medium which can reduce the acoustic energy significantly. Therefore, attenuation of the acoustic signal must be considered carefully before treatment with acoustic-wave range verification. Accurate detector positioning is also important for the same reason. For instance, if we assume that the *in vivo* attenuation coefficient is 0.54 dB/cm/MHz^28^, a 5 mm positioning error produces a range detection error of 0.16–0.88 mm (*R*_*res*_ = 1–9 mm).

Beam spot position error also causes range detection error. Assuming that the maximum spot position error during beam delivery is 0.2 mm, which occurs in current treatment machines with implemented position monitors, the deposited energy in the gold marker has a maximum uncertainty of 3.9%. This uncertainty is equivalent to an error range of 0.07–0.36 mm (*R*_*res*_ = 1–9 mm). In general, the influence of the spot position error is large for small-sized spots. For a clinical proton beam, the spot size near the Bragg peak is greater than the value used in the current study (σ = 5 mm). On the other hand, this effect requires analysis for heavy-ion beams with smaller spot sizes, such as for carbon beams.

In one perspective, the gold marker considered in this study is a possibly new type of *in vivo* point dosimeter. Previous studies have proposed several *in vivo* dosimeters such as implanting wireless dosimeters^29^ and PET imaging of radioactive markers^30^, and based on the literature, their range detection error is approximately 1 mm. Therefore, we expect that the present method should yield an equivalent range detection performance. On the other hand, a common limitation for these implanting dosimeters is that the range is determined for only a limited number of beams that pass through the dosimeters.

As demonstrated above, we estimated that the range detection error for the proposed method is less than 1 mm. However, the analysis was limited to a single condition. To establish this method’s utility, future systematic studies are required. Simultaneously, increasing the SNR is important when attempting to expand this method’s applicable range. Several approaches may be considered to do so. Large area detectors may effectively suppress any influence from thermal noise as noise amplitude is inversely proportional to the area of the PZT element^18^. The installation of multiple detectors can also reduce the influence of thermal noise. Previous studies have also explored the array type detector^31–33^. Signal averaging can be used for noise reduction if the detectors are arranged on a spherical surface around the gold marker.

A specialized transducer may further reduce the SNR. High Q-value transducers with a thin backing layer have a higher sensitivity at similar resonance frequencies than commercially available broadband transducers^34^ and are therefore suitable for application in the present study.

A shorter beam pulse and larger markers could also increase the SNR. Larger markers are more invasive, and block more of the proton dose downstream of the marker, so this option is not viable in practice. The development of less-invasive acoustically-detectable markers is a challenge for future work. Higher proton intensities and shorter beam pulses require advances in analytical capabilities, such as developments in laser-based proton acceleration and the Fixed-Field Alternating Gradient accelerator (FFAG), which are currently in progress. FFAG is especially a strong candidate because previous studies have shown that this technique provides the clinically required beam performance^35,36^, i.e., proton beam energy of 150 MeV, pulse width of <100 ns, and intensity of 10^8^ proton/pulse. However, delivering a spot dose with one pulse in the spot-scanning method requires further development with respect to the accurate control of the storage beam charge in the FFAG ring.

A conventional time-of-flight (TOF) approach could be used for the analysis involved in range verification^11,15^. This approach would allow simultaneous detection of the positions of the marker and Bragg peak, which would increase the reliability of range measurements. To implement TOF calculations in practice, the transducer positions would need to be optimised so that the arrival time of the acoustic waves from the Bragg peak could be distinguished from that generated from the marker.

Finally, comparison with previous studies is discussed. Little research in the literature has focused on a similar arrangement of instrumentation to that in our study, in terms of beam pulse parameters and detector positions. Ahmad *et al*. employed similar settings in simulations, with incident beam energy = 100 MeV, pulse duration = 100 ns and spot size = 10 mm^18^. They reported that 100 mPa pressure was generated with 2 cGy irradiation dose. Though Ahmad *et al*. did not give the same level of detail about their simulation settings, we found that 2.9 × 10^6^ particles are required to deliver a 2 cGy dose at the Bragg peak in our simulation, and this pulse generates 104 mPa of pressure. The result is therefore consistent with Ahmad *et al*.

In summary, we proposed a new range verification method using the acoustic wave generated from a spherical gold marker currently used in clinics to reduce set-up errors. Specific high-frequency acoustic waves originated from the marker were observed in numerical simulations. The wave frequency depended solely on the marker diameter, and amplitude was determined by the distance between the marker and the Bragg peak. The frequency of the resonance peak agreed well with the value derived analytically from the wave equation with the boundary condition of zero amplitude on the sphere surface, while the relation between the amplitude of the peak and initial pulse width was reproduced well in Fourier analysis. The results indicate that the measurement of the Bragg peak position may require only a single detector that is designed with consideration of the marker’s resonance frequency. Clinical implementation of the method will be easier with proton beams of higher intensity and shorter pulses, and a less-invasive acoustically-detectable marker, and these remain as challenges for future work.

## Methods

### Simulation setup

In the simulations used in this study, a spherical gold marker is placed in water, and a proton beam impinges upon the marker. Figure 1 diagrams the simulated system. A spherical gold marker is placed in a water phantom at a depth of *Z*_*m*_. The diameter of the gold sphere marker was 2.0 mm, which is typically used in current clinical practice. The proton pencil beam is incident on the water phantom along the *z*-axis, which penetrates the centre of the marker. The *x* and *y*-axes are taken as the transverse coordinates. The beam energy is 60 MeV and corresponds to proton range of about 31 mm in water. This energy is close to the minimum energy provided by accelerators for proton therapy (~70 MeV) and was chosen to reduce the amount of memory required for the wave-propagation computations. The dose at the Bragg peak is 0.7 × 10^−6^ cGy/proton in the case. Both calculations, of dose distribution and pressure wave propagation, require spatial discretisation. The calculation grid size was set to 0.2 × 0.2 × 0.2 mm^3^ in these simulations to properly represent the shape of a mm-sized spherical marker and to accurately simulate the propagation of high-frequency waves. To measure time-resolved pressure waves, point-like acoustic detectors are spaced at 0.2 mm intervals at 20.6 mm from the beam axis. 254 detectors are included in the simulation. In the wave propagation simulations, a perfectly matched layer is applied to the boundary, which absorbs all outgoing acoustic waves and prevents reflection.

### Acoustic wave equation with an external pressure source

The proton beam is injected in a very short period in which stress confinement and thermal confinement are both fulfilled; the proton pulse width *σ*_*p*_ is assumed to be much smaller than the stress and thermal relaxation times, *t*_*s*_ and *t*_*th*_ respectively^37^. Here, *t*_*s*_ and *t*_*th*_ are expressed as

9 $${t}_{s}=\frac{{d}_{c}}{v},\,{t}_{th}=\frac{{d}_{c}^{2}}{{\alpha }_{th}},$$

where *d*_*c*_ denotes the characteristic dimension of the heated region (lateral beam size of ~5 mm); *v* and *α*_*th*_ are the sound velocity and the thermal diffusivity, respectively. The values of these parameters are listed in Table 1. From *v* and *α*_*th*_ for water, *t*_*s*_ and *t*_*th*_ were calculated as 3.3 μs and 170 μs, respectively.

Under the conditions of stress and thermal confinement, the initial pressure rise due to the transferred energy can be described as follows:

10 $${p}_{0}(\overrightarrow{r},\,t)=\Gamma E(\overrightarrow{r},\,t).$$

$E(\overrightarrow{r},\,t)$ and ${p}_{0}(\overrightarrow{r},\,t)$ are the cumulative energy and the amplitude of the external pressure source at time *t*, respectively. *Γ* is the Gruneisen coefficient, which is a dimensionless quantity that describes the conversion of thermal energy to volumetric expansion and is calculated using the equation below:

11 $$\Gamma =\frac{\beta {v}^{2}}{{C}_{p}}.$$

Here *β* is the coefficient of volumetric thermal expansion and *C*_*p*_ is the heat capacity at constant pressure. Note that *Γ* for gold is ~32 times and *ρ* is ~19 times higher than those values for water. This suggests that gold markers will act as strong pressure sources during proton beam therapy.

Given the initial pressure rise ${p}_{0}(\overrightarrow{r},\,t)$, the temporal and spatial propagation of the pressure wave, $p(\overrightarrow{r},\,t)$, can be described by the following wave equation:

12 $${\nabla }^{2}p(\overrightarrow{r},\,t)-\frac{1}{{v}^{2}}\frac{{\partial }^{2}p(\overrightarrow{r},\,t)}{\partial {t}^{2}}=-\,\frac{1}{{v}^{2}}\frac{\partial }{\partial t}(\frac{\partial {p}_{0}(\overrightarrow{r},\,t)}{\partial t}).$$

The wave equations (Eq. (12)) were solved using the k-wave toolbox in Matlab^38^. The time step was set to 30 ns to ensure stability (Courant–Friedrichs–Lewy number <1). The temporal and the spatial parts of $E(\overrightarrow{r},\,t)$ are assumed to be separable as

13 $$E(\overrightarrow{r},\,t)=E(\overrightarrow{r})G(t).$$

and the beam’s pulse structure is assumed to be Gaussian, namely:

14 $$\frac{\partial G(t)}{\partial t}=\frac{1}{\sqrt{2\pi {\sigma }_{p}^{2}}}\exp \,[-\frac{{t}^{2}}{2{\sigma }_{p}^{2}}].$$

Then the right-hand side of the wave equation (12) becomes

15 $$-\frac{1}{{v}^{2}}\frac{\partial }{\partial t}(\frac{\partial {p}_{0}(\overrightarrow{r},\,t)}{\partial t})=-\,\frac{\Gamma }{{v}^{2}}E(\overrightarrow{r})\frac{{\partial }^{2}G(t)}{\partial {t}^{2}}.$$

The energy transferred by protons, $E(\overrightarrow{r})$, was calculated using the Geant4 Monte Carlo simulation software (version 9.3)^39^. To achieve sufficient statistics, 10^7^ proton histories were tracked, and the transferred energy was calculated in terms of voxels. The lateral beam profile was assumed to be Gaussian with *σ* = 5 mm at the water surface. This condition can be met by proton-therapy machines currently in clinical use. We used Geant4’s Standard Physics List option 3 configuration for the electromagnetic processes, and the G4BinaryCascade and G4HadronElastic models for the inelastic and elastic hadron processes, respectively. The cut-off range for the secondary electrons, positrons and photons was 1 mm.

### Simulation parameters

The following parameters were varied to understand their effect on the pressure waves: the width of beam pulse *σ*_*p*_ and the marker position *Z*_*m*_. The width of beam pulse *σ*_*p*_ was varied from 30 to 500 ns. To obtain a clear signal, the pulse width was chosen in the < *μs* range^18,19^, which fulfils the confinement conditions. Moreover, *Z*_*m*_ was varied from 16 to 38 mm, which corresponds to a residual range of 15 mm and −7 mm for 60-MeV protons, respectively; the marker was therefore positioned at both pre- and post-Bragg peak regions in the tests.
